# Four ways to ventilate during cardiopulmonary resuscitation in a porcine model: a randomized study

**DOI:** 10.1186/s13049-016-0262-z

**Published:** 2016-05-10

**Authors:** Benedict Kjærgaard, Egidijus Bavarskis, Sigridur Olga Magnusdottir, Charlotte Runge, Daiva Erentaite, Jes Sefland Vogt, Mette Dahl Bendtsen

**Affiliations:** Biomedical Research Laboratory, Department of Clinical Medicine, Aalborg University Hospital, Aalborg, Denmark; Department of Cardiothoracic Surgery, Aalborg University Hospital, Aalborg, Denmark; Department of Cardiothoracic Surgery, Blekinge Hospital, Karlskrona, Sweden; Elective Surgery Centre, Silkeborg Regional Hospital, Silkeborg, Denmark; Danish Armed Forces, Health Services, Aarhus, Denmark; Department of Pathology, Aalborg University Hospital, Aalborg, Denmark; Department of Gastrointestinal Surgery, Aalborg University Hospital, Aalborg, Denmark; Department of Clinical Medicine, Aalborg University, Aalborg, Denmark

**Keywords:** Ventilation methods, Cardiac arrest, CPR

## Abstract

**Background:**

The optimal method for out-of-hospital ventilation during cardiopulmonary rescue (CPR) is controversial.

The aim of this study was to test different modes of ventilation during CPR for a prolonged period of 60 min.

**Methods:**

Pigs were randomized to four groups after the induction of ventricular fibrillation, which was followed by one hour of mechanical cardiac compressions. The study comprised five pigs treated with free airways, five pigs treated with ventilators, six pigs treated with a constant oxygen flow into the tube, and six pigs treated with apnoeic oxygenation.

**Results:**

The free airway group was tested for 1 h, but in the first 15 min, the median PaO_2_ had already dropped to 5.1 kPa.

The ventilator group was tested for 1 h and still had an acceptable median PaO_2_ of 10.3 kPa in the last 15 min. The group was slightly hyperventilated, with PaCO_2_ at 3.8 kPa, even though the ventilator volumes were unchanged from those before induction of cardiac arrest.

In the group with constant oxygen flowing into the tube, one pig was excluded after 47 min due to blood pressure below 25 mmHg. For the remaining 5 pigs, the median PaO_2_ in the last 15 min was still 14.3 kPa, and the median PaCO2 was 6.2 kPa.

The group with apnoeic oxygenation for 1 h had a resulting median PaO_2_ of 10.2 kPa and a median PaCO_2_ of 12.3 kPa in the last 15 min.

**Discussion:**

Except for the free airway group, the other methods resulted in PaO_2_ above 10 kPa and PaCO_2_ between 3.8 and 12.3 kPa after one hour.

**Conclusion:**

Constant oxgen flow and apnoeic oxygenation seemed to be useable alternatives to ventilator treatment.

## Background

Mechanical chest compression devices such as LUCAS and Autopulse are used if return of spontaneous circulation (ROSC) is not promptly achieved [1]. The Guidelines for Resuscitation recommend that the devices be used in special situations with prolonged cardiopulmonary resuscitation CPR, such as CPR during transport [2]. There are no clear recommendations for oxygen and carbon dioxide levels during prolonged CPR, but after ROSC, both hyperoxia and hypocapnia seem to be harmful [3].

Bystanders often initiate compression-only CPR until the arrival of trained persons who can perform rescue breaths. After the arrival of professionals and if tracheal intubation is achieved, continuous ventilation with a rate of 10 breaths per minute is recommended, but there is still a risk of hyperventilation. After cardiac arrest, the metabolism is presumably disturbed or is anaerobic to some degree because of poor circulation with less carbon dioxide production, resulting in hypocapnia during ventilation and worse clinical outcomes [4–6].

Compression-only CPR appears to be of very limited value for more than a short time. Some ventilation occurs by chest compressions alone, provided the airways are free, but if CPR is needed for a longer time, animal experiments indicate that this is not sufficient [7, 8].

A ventilation method previously described insufflates a constant flow of oxygen into a so-called Boussignac endotracheal tube [9–11], a multichannel endotracheal tube with capillaries in the wall of the tube used for insufflation of oxygen while the tube is left open to the atmosphere.

One method of securing high oxygenation and avoiding hypocapnia can be with apnoeic oxygenation, which in both animal experiments and in humans has been shown to give very high oxygenation for many minutes without CO_2_ excretion. It is important to use pure oxygen because of the risk of accumulating nitrogen in the lungs with sudden desaturation [12–15].

During CPR, the interaction between cardiac compressions and ventilation may cause histopathologic injuries in the lungs. In an animal study with continuous compressions and ventilation, the injuries were more profound than in a 30:2 mode [16]. Theoretically, the different methods of ventilation can, themselves, have harmful effects on the lung tissue.

The standard ventilation with which we compared only free airways, continuous oxygen insufflation, and apnoeic oxygenation was continuous ventilation with the same volume settings as were appropriate before cardiac arrest. The chosen standard was close to the recommendation for advanced life support, but with an inspiratory oxygen fraction of 0.6, in an attempt to inhibit the formation of interfering atelectases.

The aim of the study was to compare continuous ventilation with the other three methods in a prolonged period of one hour of CPR.

## Methods

The Danish Animal Experiments Inspectorate, no 2013-15-2934-00944, approved the experiments. The experiments were in compliance with the Utstein-style guidelines for reporting of laboratory cardio pulmonary rescue (CPR) research [17]. The study was carried out with 24 female Danish Landrace-Yorkshire pigs (30–35 kg).

### Animal care

The animals were anaesthetised with Zoletil, a mixture of two dissociative anaesthetics (ketamine 6.25 mg/ml and tiletamine 6.25 mg/ml); a benzodiazepine (zolazepam 6.25 mg/ml); a synthetic opioid (butorphanol 1.25 mg/ml); and xylazine (6.5 mg/ml), an alpha 2 adrenergic agonist, which contains both sedative, hypnotic, analgesic and muscle relaxing properties. Just before the induction of ventricular fibrillation, the animals were paralysed with 50 mg of rocuronium to inhibit a potential confounding effect of gasping [11].

The trachea was intubated with a 6.5 mm cuffed endotracheal tube, and the lungs were mechanically ventilated with a ventilator (Dameca DREAM, Roedovre, Denmark) with tidal volume of 10 ml/kg and positive end-expiratory pressure (PEEP) of 5 cm H_2_O. The tidal volume is higher than that recommended for humans but is what is normally used for pigs [18, 19]. The respiratory rate was adjusted to keep PaCO_2_ at 4.5–5.5 kPa. The fraction of inspired oxygen was 0.23, the lowest possible set point for the ventilator, before cardiac arrest.

A catheter was inserted into a femoral artery for continuous blood pressure monitoring and for sampling of blood for analyses of PaO_2_, pH, PaCO_2_, P-potassium, and lactate (ABL 800, Radiometer, Copenhagen, Denmark). A central venous catheter was inserted via the jugular vein for drug and fluid administration. During the one-hour interval of the experiment, two litres of isotonic NaCl were infused. A bladder catheter was inserted for urine drainage.

During cardiac compressions, a continuous infusion of norepinephrine (100 mg/l, 10–50 ml/h) was administered and adjusted after 30 min as needed to keep the systolic blood pressure above 50 mm Hg. The infusion rate was adjusted in intervals of 10 ml/h, no higher than necessary to keep blood pressure above 50 mm Hg, and was, to some extent, up to the discretion of the team.

ECG, blood pressure, and end tidal CO_2_ were monitored continuously. Arterial blood was collected every 5–10 min for analysis.

### Experimental protocol

**Fig. 1:**
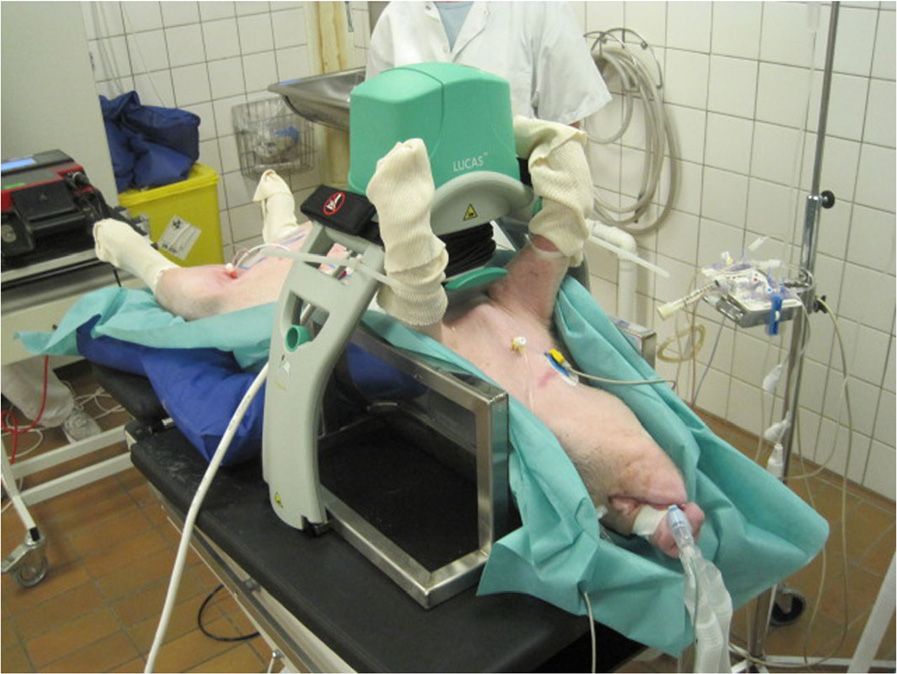
A specially constructed pig holder for the LUCAS device, which kept the pig securely positioned and slightly turned on the right side

The animals were placed in a specially constructed pig holder for the LUCAS device, keeping the pig securely positioned and slightly turned to the right side, which likely results in fewer injuries to the pig during cardiac compressions (Fig. 1).

Just before the induction of ventricular fibrillation, the animals were randomized to one of the four groups with different modes of ventilation during cardiac arrest. Ventricular fibrillation was induced using a pacemaker wire inserted through the central vein catheter into the right ventricle. A 9 volt DC shock easily provoked ventricular fibrillation. Cardiac arrest was defined as systolic blood pressure below 25 mm Hg [17].

Group 1:

Ventilation was continued using the ventilator with the same tidal volume and respiratory rate as before cardiac arrest with a fraction of inspired oxygen of 0.6 and no PEEP. The reason for not testing pure oxygen, which is recommended for clinical situations, was the theoretical risk of provoking atelectasis during one hour of ventilation.

Group 2:

The pigs had free airways due to the tracheal tube, but ventilation was induced only by chest compressions.

Group 3:

The pigs had free airways and a 10 French catheter inserted into the lower end of the tube, delivering a constant oxygen flow of 10 litres per minute. In a preceding laboratory study with the same size tracheal tube and catheter inserted into a closed box, we found that the pressure in the box was 3 cm H_2_O above atmospheric pressure, with a flow of 10 litres of oxygen into the tube. The reason for choosing this solution instead of a Boussignac tube was that in an ambulance, a 10 French catheter or something similar is typically available as a suction catheter.

**Fig. 2:**
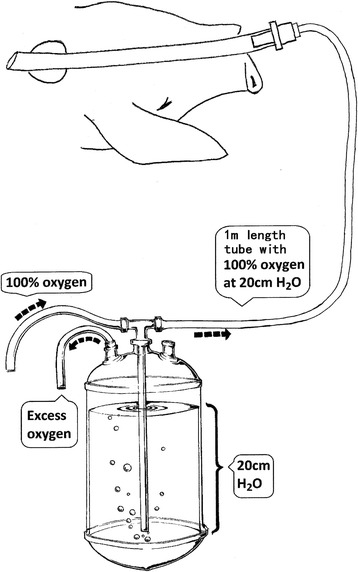
During apnoeic oxygenation, pure oxygen is delivered under controlled low pressure

Group 4:

In the group with apnoeic oxygenation, the airways were connected to a system with 100 % oxygen at a pressure of 20 cm water (Fig. 2).

For all groups, cardiac compressions were continued for one hour. If systolic blood pressure fell below 50 mmHg, a continuous infusion of noradrenalin was started but not before 30 min after onset of cardiac arrest. If the blood pressure could not be kept above 25 mmHg, the experiment was ended.

After one hour of chest compressions, the pig was euthanized with an overdose of pentobarbital. The chest was opened to harvest tissue samples from both lungs.

### Exclusion criteria

If the systolic blood pressure (SBP) dropped below 25 mmHg within 10 min after LUCAS was started, the animal was excluded due to suspicion of improper positioning of the device leading to massive intrathoracic bleeding unrelated to the ventilation.

### Histopathology

From each lung, tissue samples from the base and from the apex were examined for injuries and atelectases. All samples were taken from outside of the central area, where the LUCAS could have injured the tissue due to direct compressions of the lungs. The specimens were initially immersion-fixed in 10 % buffered formalin and subsequently embedded in paraffin. A histopathological examination was performed on 4-μm tissue sections cut from the lung samples. The samples were stained with haematoxylin-eosin. The pathologist was blinded to the modes of ventilation.

Injuries to the lung parenchyma were classified as either none; mild, if there was minor bleeding in the intraalveolar space; or severe, if there was more widespread subpleural bleeding.

The lungs were also classified as with or without atelectases. This was a rough classification, as the aim of the study was not a detailed examination of lung injuries in a pig model.

## Statistics

The number and exact timing of blood samples varied for each pig; therefore, we decided to divide the time period after cardiac arrest into the following intervals: [5; 15], [15; 30], [30; 45], and [45; 60] minutes.

The primary outcome was PaO_2_ and PaCO_2_. For O_2_ and CO_2_, the differences between the measurements before cardiac arrest and the average values of the first and second intervals were calculated, and the non-parametric Kruskal-Wallis test was applied to evaluate the null hypothesis that several samples were from the same population. Dunn’s test with Bonferroni correction was applied as a post hoc test to identify which group differed from each other.

A value of *p* < 0.05 was regarded as statistically significant.

Stata Version 13.1 (Stata Corporation, College Station, TX, USA) was used for all calculations and graphs.

## Results

After the exclusion of two pigs due to improper cardiac compressions with massive bleeding from damage to the left lung, the study comprised 22 pigs: 5 pigs treated with a ventilator, 5 pigs treated with free airways, 6 pigs treated with a constant oxygen flow, and 6 pigs treated with apnoeic oxygenation.

In the ventilator group, all 5 pigs were tested for 60 min. In the last interval, median PaO_2_ was still 10.3 (9.6–41.4) kPa. There was already a decrease in median PaCO_2_ to 3.7 (3.7–4.3) kPa in the first interval, and the level was almost constant for the rest of the experiment. At the end of the period, the median SBP was 44 (43–74) mm Hg. Three pigs were treated with norepinephrine.

In the group with free airways, all 5 pigs were tested for 60 min. In contrast to the other groups, all pigs developed a rapid decrease in oxygenation within the first interval to a median PaO_2_ of 5.1 (2.2–10.4) kPa. In the last interval, blood pressure was only median 35 (33–72) mm Hg, and 3 of the pigs needed norepinephrine to obtain this level.

In the group with constant oxygen flow, 5 of 6 pigs were tested for 60 min; in one pig, the SBP could not be kept above 25 mm Hg for more than 47 min. The pig with low SBP also had a low PaO_2_ throughout the experiment. In the other 5 animals, the median PaO_2_ was 14.3 (4.3–67.8) kPa in the last interval, PaCO_2_ was median 6.2 (5.1–17.6) kPa, and the BP was median 69 (33–75) mm Hg; 3 pigs were treated with norepinephrine.

In the group with apnoeic oxygenation, all 6 pigs were tested for 60 min. In the last interval, PaO_2_ was median 10.2 (7.1–58.2) kPa, PaCO_2_ was median 12.3 (8.2–21.6) kPa, and the SBP was median 54 (37–76) mm Hg. One pig was treated with norepinephrine.

In the first 15 min, Kruskal-Wallis test already showed that the values of PaCO_2_ could not come from the same population (*p* < 0.001), while the test for PaO_2_ passed below a *p*-value of 0.05 in the second quarter (Table 1).

**Table 1:** The first part of the table displays Kruskal-Wallis test results regarding differences between groups in the development of PaO2 and PaCO2, respectively, from before cardiac arrest to both the first and the second quarter. The second part of the table displays the bonferroni corrected *p*-values from pairwise comparisons between groups performed by the Dunn’s post hoc test regarding PaO2 and PaCO2

	PaO2		PaCO2	
	Before cardiac arrest vs. first quarter	Before cardiac arrest vs. second quarter	Before cardiac arrest vs. first quarter	Before cardiac arrest vs. second quarter
Kruskal-Wallis test	*p* = 0.087	*p* = 0.051	*p* = 0.001	*p* < 0.001
Dunn’s post hoc test				
Ventilator vs. Apneic oxygenation	*p* > 0.999	*p* = 0.170	*p* < 0.001	*p* < 0.001
Ventilator vs. Free airways	*p* = 0.154	*p* = 0.058	*p* = 0.039	*p* = 0.034
Ventilator vs. Constant oxygen flow	*p* > 0.999	*p* > 0.999	*p* = 0.363	*p* = 0.270
Constant oxygen flow vs. Apneic oxygenation	*p* > 0.999	*p* > 0.999	*p* = 0.049	*p* = 0.043
Constant oxygen flow vs. Free airways	*p* = 0.066	*p* = 0.062	*p* = 0.891	*p* > 0.999
Free airways vs. Apneic oxygenation	*p* = 0.113	*p* = 0.069	*p* = 0.638	*p* = 0.501

Except for in the free airway only group, the values of PaO_2_ were higher than 10 kPa until the end of the experiment.

Dunn’s test performed for PaCO_2_ values only revealed no difference between the ventilator group and the constant oxygen flow group. Differences between the ventilator group and the free airway only group and apnoeic oxygenation group were significant from the first quarter, *p* < 0.05 and *p* < 0.01, respectively.

**Fig. 3:**
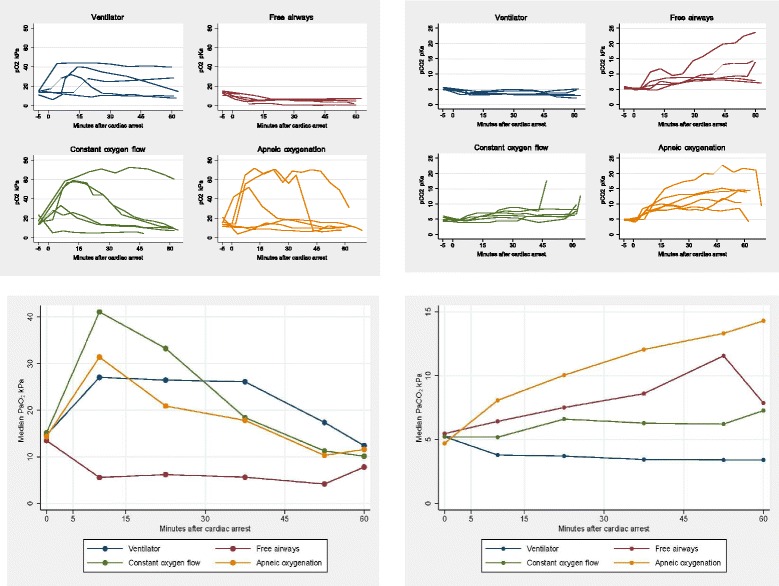
Arterial oxygen and carbon dioxide tension during 60 minutes of cardiac compressions for all animals and median arterial tensions for each 15 minutes of cardiac compressions

Figure 3 shows the values of arterial oxygen and carbon dioxide tension in the 4 groups. Figure 3 and Table 2 also show the median of the average values of PaO_2_ and PaCO_2_ for each 15 min interval of cardiac compressions.

**Table 2:** Median arterial oxygen and carbon dioxide tensions for each 15 minutes of cardiac compressions

PaO_2_ kPa	Before cardiac arrest median (min - max)	[5 min. 15 min[median (min - max)	[15 min. 30 min[median (min - max)	[30 min. 45 min[median (min - max)	[45 min. 60 min[median (min - max)
Ventilator	14.7 (11.2–15.6)	27.0 (12.1–44.3)	20.2 (9.7–44.2)	11.8 (10.2–41.8)	10.3 (9.6–41.4)
Free airways	13.5 (11.1–15.2)	5.1 (2.2–10.4)	6.2 (1.5–7.1)	4.5 (0.7–6.9)	3.1 (1.1–6.8)
Constant oxygen flow	15.2 (13.4–23.5)	41.1 (6.2–60.4)	33.2 (4.9–69.6)	12.9 (5.8–69.9)	14.3 (4.3–67.8)
Apneic oxygenation	14.4 (11.7–21.5)	31.4 (8.1–68.2)	15.4 (8.5–69.1)	18.0 (6.7–68.9)	10.2 (7.1–58.2)
PaCO_2_ kPa	Before cardiac arrest median (min - max)	[5 min, 15 min[median (min - max)	[15 min, 30 min[median (min - max)	[30 min, 45 min[median (min - max)	[45 min, 60 min[median (min - max)
Ventilator	5.2 (5.0–5.7)	3.7 (3.7–4.3)	3.7 (3.4–4.8)	3.5 (3.3–4.6)	3.8 (2.6–4.4)
Free airways	5.5 (5.2–6.1)	6.4 (4.7–11.3)	7.5 (7.0–11.3)	10.9 (8.2–17.9)	13.8 (8.7–21.3)
Constant oxygen flow	5.2 (4.2–6.1)	5.2 (3.9–6.6)	7.0 (4.3–7.9)	6.6 (4.6–8.2)	6.2 (5.1–17.6)
Apneic oxygenation	4.7 (4.6–5.2)	8.1 (7.5–9.4)	9.6 (8.3–16.2)	11.0 (8.5–19.2)	12.3 (8.2–21.6)

**Fig. 4:**
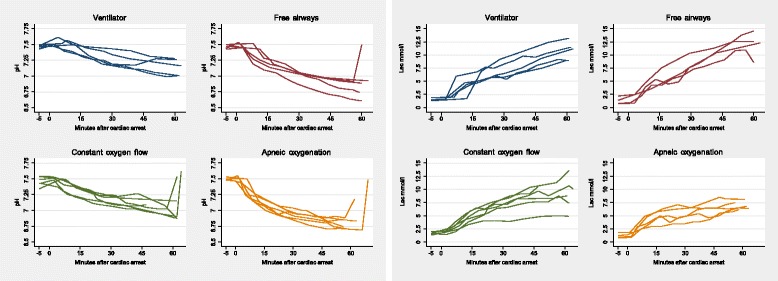
Arterial pH and plasma lactate during 60 minutes of cardiac compressions for all animals

For all groups, the pH decreased and p-lactate increased during the 60 min of CPR. The least affected values were observed in the group treated with the ventilator and in the group treated with constant oxygen flow (Fig. 4).

**Fig. 5:**
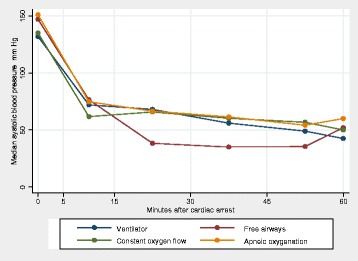
Systolic blood pressure during 60 minutes of cardiac compressions

Figure 5 shows the median of the average blood pressure in the 4 groups. The blood pressure decreased in all groups. This seemed to be less pronounced in the groups with apnoeic oxygenation and with constant oxygen flow.

### Histopathology

Three pigs were excluded from the histological examinations because of technical problems with the conservation, reducing the number of animals with a histological examination to 5 pigs in the free airway group, 5 pigs in the ventilator group, 5 pigs in the constant oxygen flow group, and 4 pigs in the group with apnoeic oxygenation.

The histological changes seemed to be less pronounced than those described by Wang et al. [16]. Overall, the histological changes seemed to be less pronounced in the group with apnoeic oxygenation and most pronounced in the group with free airways only, but the sample sizes were too small for a statistical evaluation (Table 3).

**Table 3:** Histopathological changes in lung parenchyma

Histology	No changes	Mild changes	Severe changes	Atelectases
Free airways	1	1	3	3 of 5
Ventilator	2	2	1	1 of 5
Constant oxygen flow	1	3	1	3 of 5
Apneic oxygenation	2	2	0	1 of 4

## Discussion

All groups except the free airway only group had a PaO_2_ of at least 10 kPa during 60 min of CPR. In the free airway only group, the oxygenation fell rapidly to a dangerous level. In hands on only CPR, the patient will normally not be intubated and therefore does not have a secure airway. All ventilatory methods except for apnoeic oxygenation could handle excretion of CO_2_ but an unaltered ventilator volume setting resulted in slight hyperventilation. Apnoeic oxygenation gave high oxygenation but during 60 min of CPR, PaCO_2_ rose to 12.3 (8.2–21.6) kPa.

A likely reason for low PaCO_2_ in the ventilator group may be decreased circulation with anaerobic metabolism in parts of the body and consequent reduced carbon dioxide excretion. It has been shown in several articles that hyperventilation is common in CPR and that mortality is higher when the patient is hyperventilated [4–6]. What may contradict the hypothesis that hyperventilation per se is the cause of higher mortality is the observation of lower carbon dioxide excretion in cases with poor body circulation, which will cause a lower PaCO_2_ even though the ventilation rate is unchanged.

The group with free airways only also seemed to have more pronounced histological changes in the lungs, although the number of animals was too small for statistical conclusions.

The group treated with constant oxygen flow had an acceptable PaO_2_ after 60 min, but there was a tendency towards greater variation in the PaO_2._ PaCO_2_ remained stable at a slightly higher level than in the ventilator group. It is likely that the level of carbon dioxide depends on the flow of oxygen into the tube. Our solution with a tiny catheter inserted into the tracheal tube may not work as well as the originally described Boussignac tube with its multichannel system. In sizes appropriate for humans, it has been described as resulting in a positive airway pressure of 10 cm H_2_O above atmospheric pressure if the flow was 15 litres of oxygen per minute [9], but in the actual study, we measured a pressure of only 3 cm H_2_O. The consequences of higher mean intrathoracic pressure on coronary and cerebral circulation have not been proven, but several studies have shown a tendency of a higher frequency of ROSC but not a higher survival rate in groups treated with constant oxygen flow compared to patients treated with manual ventilation [20, 21].

The group with apnoeic oxygenation maintained good saturation for 60 min but with a constant rise in PaCO_2_. To some extent, the rise in carbon dioxide may be beneficial for a period, which is in contrast to the situation with unintended hyperventilation during CPR because of the resulting shift of the oxygen dissociation curve to the right side due to acidosis. The constant higher pressure in the thoracic cage may keep the lungs open, and there seemed to be less atelectasis in the group; however, the number of pigs was too low to draw conclusions. On the other hand, a higher intrathoracic pressure may inhibit the venous return to the heart and have a negative influence on resuscitation [22, 23]. However, the blood pressure in this group was not lower than in the other groups. Performing apnoeic oxygenation in a clinical environment demands a pressure-regulated valve to supply pure oxygen with a low pressure of 5–20 cm H_2_O, which can greatly simplify the procedure after endotracheal intubation.

## Limitations

Even though the LUCAS device was originally designed using pigs, the device is for human use, and our specially constructed pig holder was not validated and may only be suitable for this specific breed of pigs. The anatomic differences between pig and human thoracic cages may limit a translation of the results to the humans.

The study could be performed only with endotracheal intubation, which could give even better results than in hands on only CPR for the group with free airways only.

To some extent, norepinephrine treatment at the discretion of the team could contribute to bias, and the study was not designed for testing the best blood pressure during cardiac compressions in combination with different ventilation modes.

## Strengths

In this animal study, it was possible to achieve randomization and to create a controlled cardiac arrest. It was possible to extend the period of CPR to a longer duration than normal in an attempt to find the limits of the treatments through continuous testing of arterial blood gases.

## Conclusion

In this experiment with cardiac compressions after induced ventricular fibrillation, animals were randomized to one of four ventilatory methods, either ventilator treatment with FiO2 0.6 and the same volumes as before cardiac arrest, free airways only, constant oxygen flow into the tracheal tube or to apneic oxygenation.

Except for the group with free airways only, the other methods all provided acceptable oxygenation during one hour of cardiac compressions with no major injuries to the lungs. The most appropriate levels of carbon dioxide seemed to be in the group with constant oxygen flow in the tube.
